# Temporal Inflammatory Profile in Skin Surface after Oronasal Mask Application

**DOI:** 10.2340/actadv.v106.adv-2025-0159

**Published:** 2026-08-31

**Authors:** Amanda Feldt, William Lövfors, Elin Nyman, Peter R. Worsley, Bijar Ghafouri, Sara Bergstrand

**Affiliations:** 1 Department of Health, Medicine and Caring Sciences, Linköping University, Linköping, Sweden; 2 Clinical Department of Acute Internal Medicine in Linköping, Region Östergötland, Linköping, Sweden; 3 Department of Biomedical Engineering, Linköping University, Linköping, Sweden; 4 Skin Sensing Research Group, School of Health Sciences, University of Southampton, Southampton, England, United Kingdom

**Keywords:** biomarkers, inflammation, respiratory therapy, sebum, skin soft tissue injuries

## Abstract

Skin damage from routine oronasal facemask use is common, but the early molecular changes and timing of inflammatory cytokine responses remain poorly characterized due to limitations in conventional detection methods. Early pressure ulcer formation involves a critical window where physiological changes occur before visible signs. This study addresses this gap, aiming to monitor the temporal inflammatory profile in skin surface after the standard use of oronasal facemasks. Sebum samples were collected from 11 patients at 6 timepoints up to 180 min postmask removal. A panel of 71 inflammatory proteins was analysed using multivariate statistics, protein network analysis and mathematical modelling. Two distinct cytokine response phases were identified: an early phase (0–20 min) and a delayed phase (40–180 min), with 17 cytokines driving this separation. Notably, the delayed phase showed sustained elevation despite the absence of visible erythema, indicating ongoing subclinical inflammation. Network analysis revealed activation of pathways related to angiogenesis, cell death regulation and tissue remodelling – hallmarks of early wound healing and microvascular stress. These findings enhance understanding of early pressure ulcer pathophysiology, identify optimal biomarker sampling windows and support the development of point-of-care biosensors for early detection and prevention of pressure-induced skin damage.

**SIGNIFICANCE**

This study addresses important aspects of early intervention and prevention of skin tissue damage after treatment with respiratory facemasks, a common and costly complication. This study reveals previously unrecognized local inflammatory and microvascular responses following routine facemask use, offering new insights into pressure ulcer pathophysiology. By identifying precise cytokine dynamics and validating temporal clusters, it provides a new understanding for early tissue stress detection and suggests a relevant sampling window for future biomarker studies.

Skin serves as the primary barrier against external insults, crucial for overall health. The integrity of the epidermal–dermal junction is essential for maintaining homeostasis, which may be compromised by external insults, such as mechanical loading (1), potentially leading to skin tissue damage, i.e. pressure ulcer. Pressure ulcers are localized injuries to the skin and underlying tissue caused by sustained pressure or shear, typically over bony prominences. Their development involves ischaemia, lymphatic impairment and direct cellular deformation, influenced by pressure magnitude, duration and individual susceptibility (2). Acute inflammation is a rapid response to such noxious stimuli, coordinating signalling pathways to preserve tissue homeostasis (3). In response to skin tissue damage, microvascular changes occur, leading to leakage of fluids, proteins and cells into the extracellular space, visualized as the cardinal signs of inflammation; redness, swelling, pain and heat (4).

Device-related pressure ulcers from oronasal facemasks, used to improve respiratory function, are well-documented and increasingly common (5). The nasal bridge, lacking protective soft tissue, is particularly vulnerable, leading to rapid progression from visible erythema to full-thickness damage (6). A critical period exists between initial damage and visible signs, during which inflammatory changes can be objectively detected, i.e. molecular biomarkers (7). Early intervention during this window may prevent damage progression and potentially reverse the damage before it becomes irreversible.

Cytokines, which are low molecular weight proteins that regulate inflammatory pathways through a complex network of interactions (8), have shown to be potential biomarkers for pressure-induced skin tissue damage (9).  Cytokines can be collected locally from the skin surface using adhesive tape that absorbs sebum and cytokines from dermis and epidermis (10). Limited studies have shown some promising candidates such as the proinflammatory interleukin-1 (11), interleukin-15, TNF-α (12), G-CSF and IL1RA (13). However, the exploration of inflammatory biomarkers in tissue response to mechanical load has been limited by the panels offered by standard ELISA techniques. A previous explorative analyse of 71 cytokines identified 21 cytokines distinguished mechanically loaded sites from control sites (14). Baseline and forehead control measurements were also validated and confirmed local inflammatory upregulation induced by the facemask. Now, we expand on these results to explore the nature and timing of the inflammatory response and deepen the understanding of key mechanisms in inflammatory processes related to early tissue damage by developing a mechanistic mathematical model for the timing of events in the response to pressure, not previously explored.

This study aims to explore the temporal inflammatory profile in skin sebum after the standard use of oronasal facemasks in a clinical intensive care setting, using bioinformatics and mathematical modelling.

## MATERIALS AND METHODS

### Design and study setting

A repeated measurements design explored the inflammatory profile at various timepoints following skin exposure to mechanical load from standardized oronasal mask therapy. Eleven adult patients receiving their ordinary thoracic intensive care were included through convenience sampling from Linkoping University hospital in Sweden. Inclusion criteria were prescribed therapy with non-invasive ventilation masks, while the sole exclusion criterion was facial skin tissue damage.

### Sample collection and biochemical analysis

Proteins were collected from the skin surface at the nasal bridge at 6 different time intervals namely, directly after the removal of the oronasal facemask (0 min), after 20 min, 40 min, 60 min, 120 min and 180 min according to validated protocols (10, 15). A single piece of adhesive tape (Sebutape^®^, CuDerm, Dallas, TX, USA) was cut in half (~14.3 × 19.0 mm) and applied using gentle, standardized manual pressure to the marked sampling site to ensure consistent placement across repeated measurements. Tapes were removed after 2 min and stored at −80 °C. Gloves and tweezers were used throughout the entire procedure.

On the day of analysis, the tapes were thawed, and proteins were extracted by adding 300 µL phosphate-buffered saline (PBS) containing 0.05% Tween-20 and mixed gently for 1 h at 4 °C. The samples were centrifuged, and the supernatants were analysed for 71 inflammatory proteins by a U-PLEX (catalogue number K15067M) assay based on an electrochemiluminescent method (MESO QUICKPLEX SQ 120, Meso Scale Diagnostics, Rockville, MD, USA), as described in detail elsewhere (14). Standard curves were formed by fitting electrochemiluminescence signal from calibrators to a weighted 4-parameter logistic model. The DISCOVERY WORKBENCH (Meso Scale Diagnostics, Rockville, MD, USA) software was used to analyse the data. Any value that was below the lowest limit of detection (LLOD) for the assay was replaced with half of LLOD of the assay.

### Statistical analysis

Descriptive data were collected from patients’ medical records, and statistics of the characteristics of participants were analysed using SPSS^®^, version 28.0 (IBM Corp.; Armonk, NY, USA). A multivariate data analysis was used to analyse the protein data set, using the software SIMCA (version 18.0; Sartorius Stedim Biotech, Umeå, Sweden) as earlier described (16) and in accordance with Wheelock and Wheelock (17). A principal component analysis (PCA) was performed to get an overview of the protein data and to detect potential outliers, using score plot, Hotelling T2 and distance to model in X-space. Variables were mean centred and scaled for unified variance (UV-scaling). An orthogonal partial least square discriminant analysis (OPLS-DA) was used to assess differences between the different timepoints and determine which proteins were most important for separation between the timepoints. The goodness of fit (*R*^2^) is represented by the fraction of sum of squares of all the variables explained by a principal component. The goodness of prediction (*Q*^2^) is the fraction of total variation of the variables that can be predicted by a principal component using cross validation methods. These 2 model diagnosis parameters indicate if the model is robust, the *R*^2^ value should be higher than *Q*^2^ and the differences between them should be <0.3. The variable influence of projection (VIP) was used to measure the importance of each protein. P(corr) represents the loading of each variable scaled as a correlation coefficient. VIP>1 and |p(corr)|>0.4 were considered as significant.

Cross-validated analysis of variance (CV-ANOVA) was conducted for the validity of the model, a *p*-value <0.05 was considered as significant. Since the data were not normally distributed, the Mann–Whitney *U* test was conducted to test the significance level of comparison of protein expression between the timepoints. A *p-*value <0.05 was considered as significant.

### Mathematical modelling

Mathematical modelling has previously been used for studying sustained anti-inflammatory drug response (18) and mechanisms of acute inflammation in immune cells (19) also connected to clinical responses of acute inflammation, such as tissue damage (20).

To capture the dynamic behaviour of cytokines over time, mechanistic models utilizing ordinary differential equations were developed. These models aimed to elucidate the time-resolved response to mechanical loading ($u_{mask}$) with minimal structural assumptions. For the fast-responding cytokines, a single state model ($x_{cyt}$) considered cytokine production (basal production, kb, and stimulated production, ka) and degradation (kd) (Equation 1).

(1) $$\frac{dx_{cyt}}{dt}=kb+ka\times u_{mask}-kd\times x_{cyt}.$$

For the late-responding cytokines, a 2-state model ($x_{mRNA}{,x}_{cyt}$) considered cytokine production and degradation, as well as possible delays such as transcription/translation and/or signal transduction (kt) (Equation 2-3).

(2) $$\frac{dx_{mRNA}}{dt}=kb+ka\times u_{mask}-kt\times x_{mRNA},$$

(3) $$\frac{dx_{cyt}}{dt}=kt\times x_{mRNA}-kd\times x_{cyt}.$$

Model parameters (θ) were determined using experimental data by comparing the simulated model results (${{\hat{y}}_{t}(\theta )=x}_{cyt}$) with observed cytokine levels ($y_{t}$), weighted by the standard error of the mean (${SEM}_{t}$), summed over all time points in data (N), using Equation 4.

(4) $$V(\theta )=\sum_{t=1}^{N}\frac{{(y}_{t}-{\hat{y}}_{t}(\theta ))}{{SEM}_{t}}$$

To minimize the objective function, the parameter (θ) that minimizes V(θ) were searched for using optimization algorithms. Parameters were either accepted or rejected based on a $\chi^{2}$ test with N degrees of freedom and *p*=0.05. This test evaluates the agreement between observed and simulated data by using the objective function V(θ) as the test statistic.

### Bioinformatics

The bioinformatic tool STRING, version 11.5, was used to explore known and predicted protein–protein interactions and their biological processes (21). The protein accession numbers were entered in the multiple proteins search engine for the proteins most important for the model (VIP>1, |p(corr)|>0.4). The following settings were used: Organism *Homo sapiens*; interaction score was high confidence (0.70); the maximum number of interactions were only query proteins and a false discovery rate (FDR)<0.05 was used when classifying the Biological Process (GO) of each protein. In the network figure, protein–protein interaction and association are represented by a line, and each protein is represented as a coloured node. Higher combined confidence scores are represented by thicker lines.

A protein–protein interaction (PPI) enrichment *p*-value was reported.

## RESULTS

Eleven thoracic intensive care unit patients (4 women, 7 men) were included late in year 2019. The mean age was 69 ± 6 years (SD; range 57-77 years, median 69 years). All underwent thoracic surgery, primarily valve replacement (*n*=7) and/or coronary artery bypass graft (*n*=4) which led to systemic inflammation, with a mean CRP of 120±137 mg/L (SD; range= 5-362 mg/L, median=71 mg/L). All patients received an individualized prescription of non-invasive ventilation (NIV) treatment with oronasal face mask by their physician; the mean duration time was 22±8 min (SD; range=9-41 min, median=24 min). Eight patients were fitted with a preventive gel pad at the nasal bridge during the therapy session.

In this study, proteins were collected by Sebutape^®^ from the skin surface at the nasal bridge directly after NIV-therapy and sequential up to 180 min. Proteins that fell below the detection limit in more than 50% of the groups (*n*=11) were excluded from further analysis, leaving 60 proteins (of the 71 analysed). Due to patients' medical intervention needs, 9 observations were missing from the data set.

**Fig. 1.**
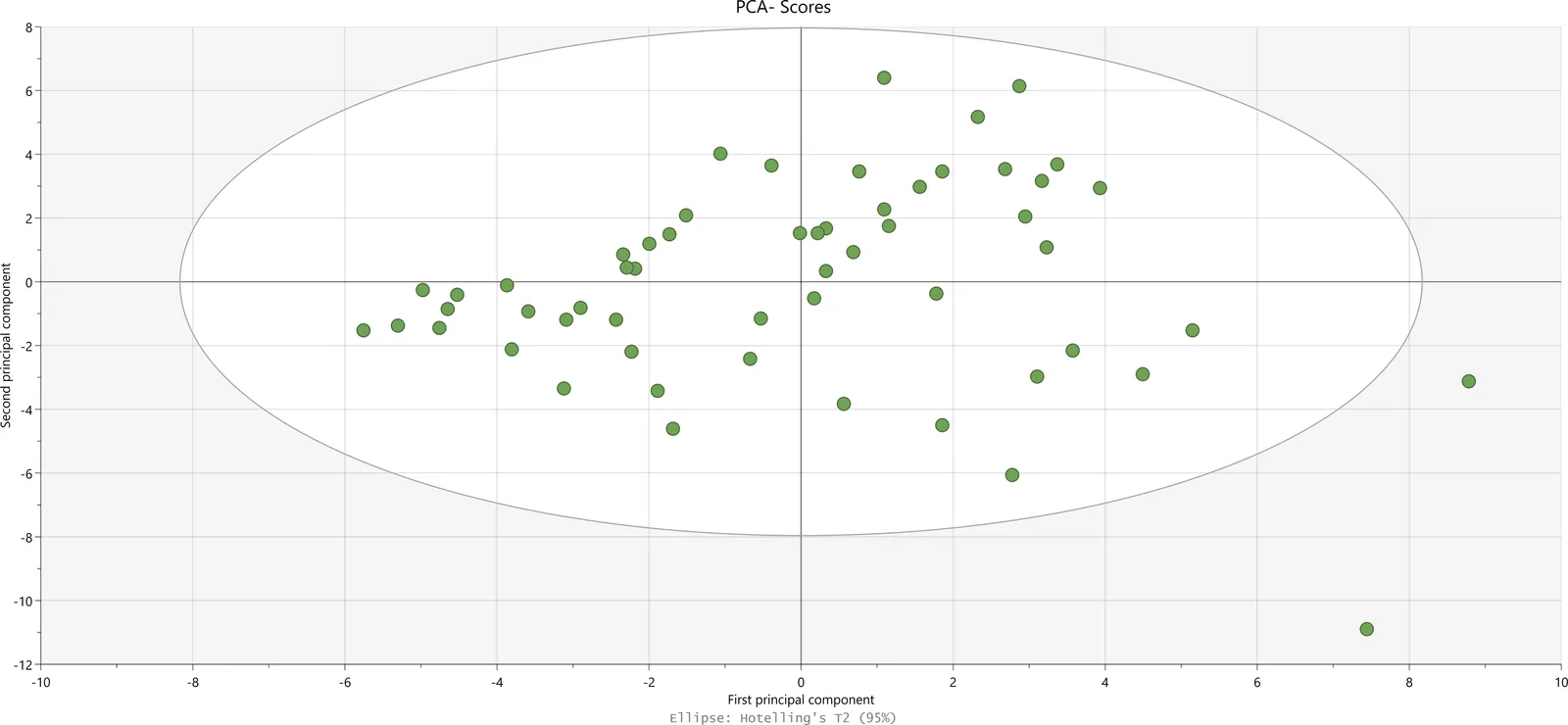
Principal component analysis (PCA) of the proteome over time. The score plot shows protein expression across all measurement time points. The scores plot scale results from the projection of the data onto the principal component. The first component (x-axis) describes the largest variation in multivariate space. The second largest variation is described by the second component (y-axis). Each point represents one sample. The ellipse represents the 95% Hotelling's T² confidence region.

The PCA identified a severe outlier, which was subsequently removed, resulting in 56 remaining observations for analysis. The new PCA including 60 proteins and 56 observations showed only moderate outliers that did not affect the model, **Fig. 1**. The data set was initially divided into 3 groups according to the timepoints of protein sampling (group 1=0 + 20 min, group 2=40 + 60 min, group 3=120 + 180 min). An autofitted orthogonal partial least square discriminant analysis (OPLS-DA) gave 1+0 components, a new component was added (1+1) which gave the model a robust value and a moderate prediction value (*R*^2^=0.362, *Q*^2^=0.187). The model showed a statistically significant (<0.0001) difference in CV-ANOVA. Since there was no difference among the later timepoints (group 2=40 + 60 min and group 3=120 and 180 min), they were merged into one group.

**Fig. 2.**
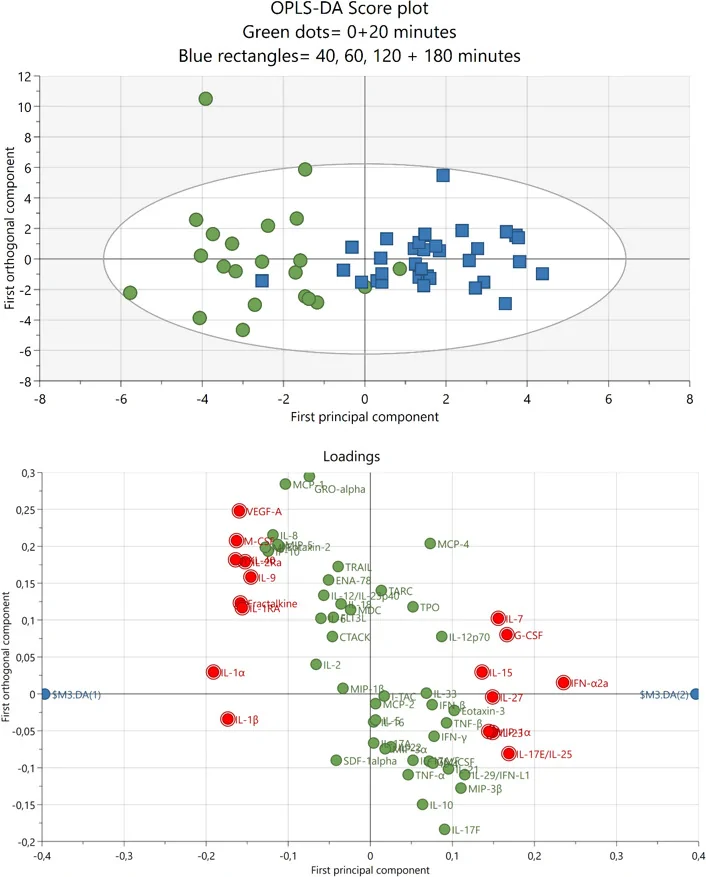
An orthogonal partial least square discriminant analysis (OPLS-DA) of the proteome over time. The x-axis represents the first predictive component, describing protein variation associated with differences between time points. The y-axis represents the first orthogonal component, reflecting individual variation within the time points. The axes do not have a physical unit; they are dimensionless. (a) The score plot shows the changes of protein expression between 2 groups: Group 1 (green dots) =0 + 20 min, group 2 (blue rectangles) =40,60,120 + 180 min). R2=0.685, Q2=0.391, CV ANOVA p-value<0.001. (b) The loading plot visualizes the protein expression associated with early (0 and 20 min; M3.DA(1)) and later (40-180 min; M3.DA(2)) time intervals. The 17 inflammatory proteins most important for separation between the 2 groups are shown as red.

The new and final OPLS-DA model with 2 groups (early and late timepoints, 0+20 min and 40, 60, 120+180 min) was conducted (2 components; 1 predictive and 1 orthogonal, *R*^2^=0.685, *Q*^2^=0.391, CV-ANOVA *p-*value<0.0001). The score plot and the loading plot of the final OPLS-DA model are visualized in **Fig. 2**. This model identified 17 proteins that contributed significantly (VIP>1.2 and │p (corr)│>0.4) to the separation between the different timepoints, Table SI.

The 17 key inflammatory proteins distinguishing the timepoints were analysed with the Mann–Whitney *U* test for comparisons. Sixteen of these proteins showed significant differences, barplots are presented in Figs S1 and S2.

**Fig. 3.**
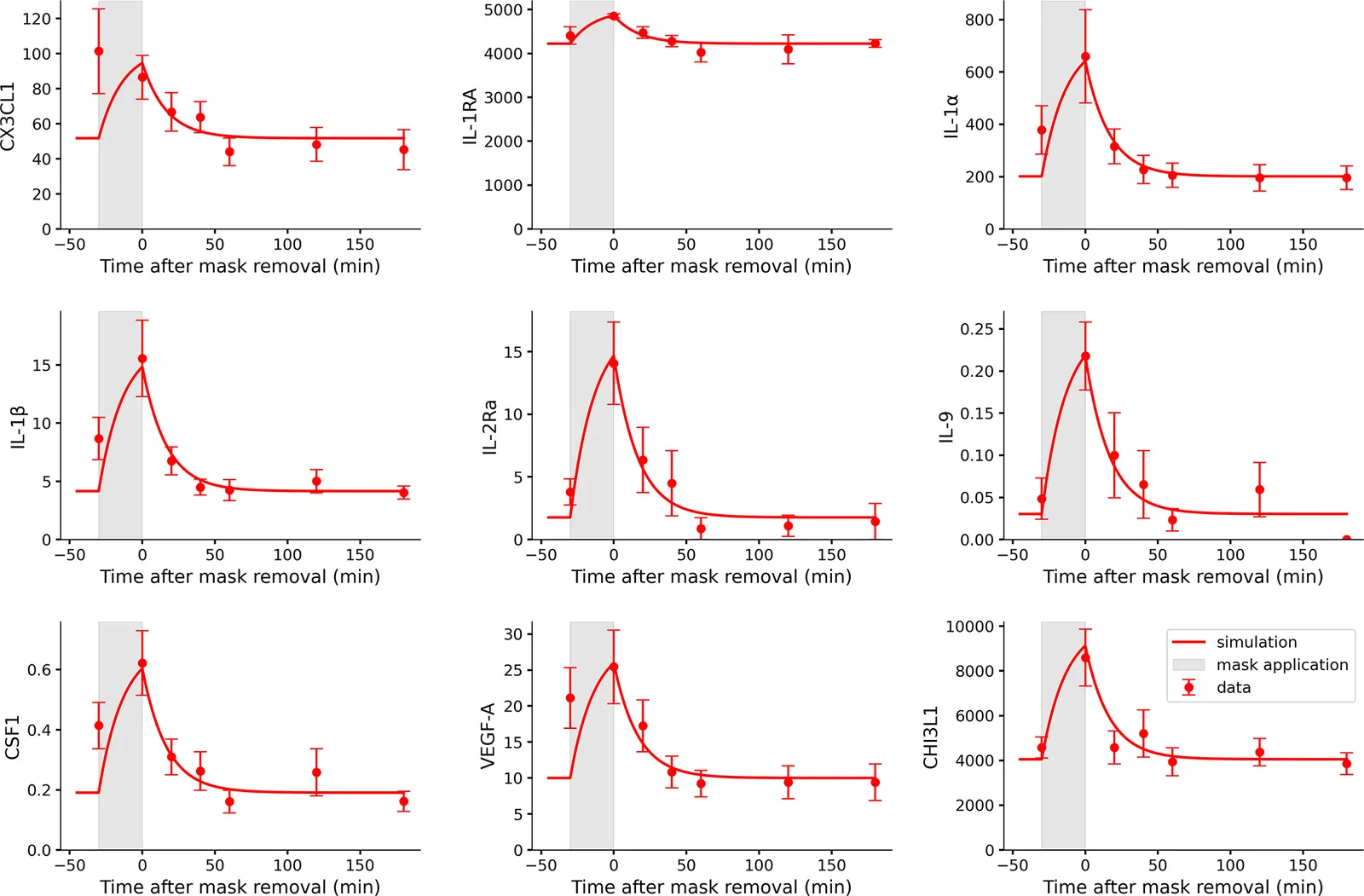
The proteins of importance for early time points show fast dynamics. The y-axis shows protein concentration (pg/mL). Time-series data (mean, dots with error bars, SEM) and mathematical model simulations (lines) for the early responding proteins. The grey area represents the time when the mask was applied, and shows the mean baseline measurement, timepoint 0, 20, 40, 60, 120 and 180 min postmask removal.

Ordinary differential equation models were developed to further explore the dynamics of the inflammatory proteins. The 17 inflammatory proteins were divided into 2 groups depending on their separation between the timepoints (early and late). The early responding proteins (*n*=9) were modelled with 3-parameter, single-state mathematical models (method section, Mathematical modelling). After parameter estimation, developed models were in agreement with the corresponding time-series data according to a $\chi^{2}$ test (**Fig. 3**). The proteins in this group increased in concentration during the mask application (grey area in Fig. 3) and decreased in concentration after the mask was removed.

**Fig. 4.**
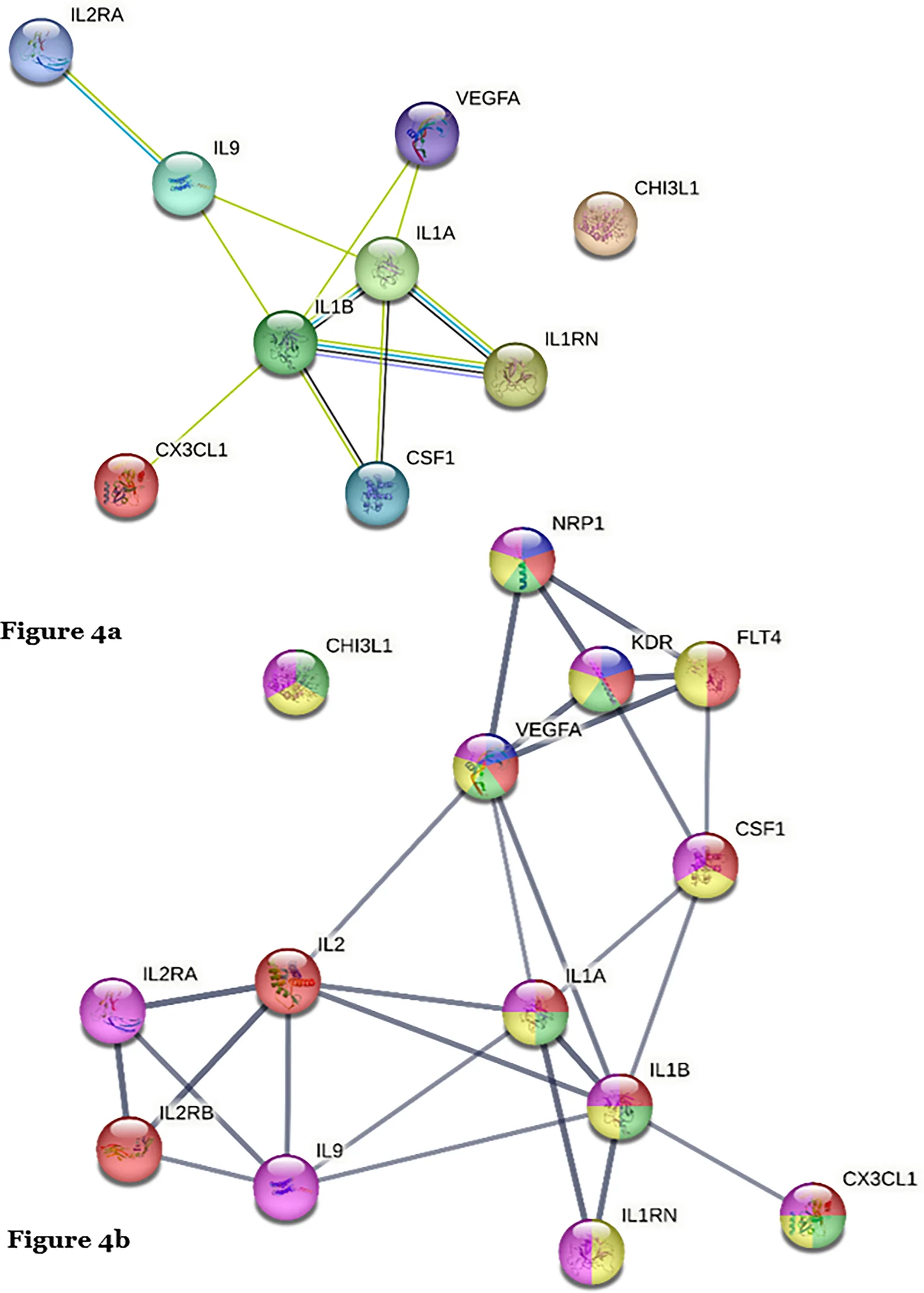
Protein network analysis of the 9 inflammatory proteins of most importance for the early timepoints. (a) A protein interaction network of the 9 inflammatory proteins of most importance in the early timepoints (0 and 20 min after loading in skin). The model has a high confidence interval (0.70), PPI enrichment value <0.0001. (b) Extended pathway analysis of the inflammatory response in early timepoints. Biological processes according to colours: Red: Angiogenesis involved in wound healing (false discovery rate (FDR) *p-*value<0.0001), purple: cell migration involved in sprouting angiogenesis (FDR *p-*value<0.0001), yellow: negative regulation of cell death (FDR *p-*value<0.0001), green: response to stress (FDR *p*-value <0.0001), blue: response to wounding (FDR *p*-value =0.0016). The model has a high confidence interval (0.70), PPI enrichment value <0.0001).

The early responding proteins were further analysed to explore interactions and biological processes in a pathway analysis. The network interaction model showed a significant protein–protein interaction (PPI) enrichment value (<0.001) as illustrated in **Fig. 4**a and the extended network in Fig. 4b. To explore larger connections, associated proteins were added into an extended network (Fig. 4b). In this model consisting of early timepoints, 5 connecting proteins were added into the network, namely, interleukin-2 receptor subunit beta (IL2RB), interleukin-2 (IL-2), vascular endothelial growth factor receptor 2 (KDR), vascular endothelial growth factor receptor 3 (FLT4) and neuropilin-1 (NRP1). The biological processes involved in the network included angiogenesis involved in wound healing (false discovery rate (FDR) *p-*value<0.0001), cell migration involved in sprouting angiogenesis (FDR *p-*value <0.0001), negative regulation of cell death (FDR *p-*value <0.0001), response to stress (FDR *p*-value <0.0001) and response to wounding (FDR *p*-value =0.0016).

**Fig. 5.**
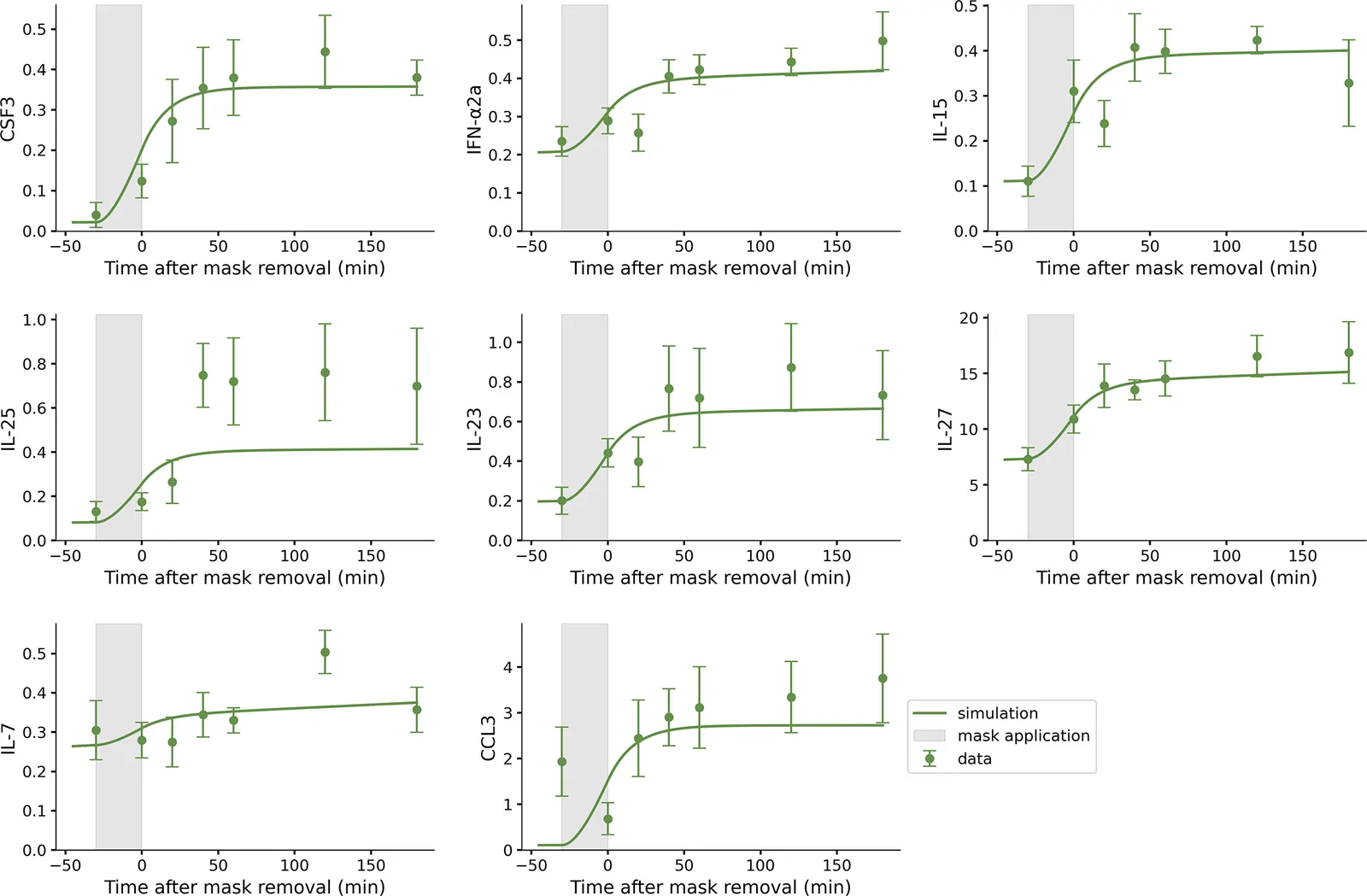
The proteins of importance for late time points show slower dynamics and a new steady state. The y-axis shows protein concentration (pg/mL). Time-series data (dots with error bars) and mathematical model simulations (lines) for the late responding proteins. The grey area represents the time when the mask was applied, and shows the mean baseline measurement, timepoint 0, 20, 40, 60, 120 and 180 min post-mask removal.

The late responding proteins (*n*=8) were modelled with 4-parameter, 2-state mathematical models (method section, Mathematical modelling). After parameter estimation, all developed models but the IL25 model were in agreement with the corresponding time-series data according to a $\chi^{2}$ test (**Fig. 5**). The proteins in this group increase in concentration during the mask application (grey area in Fig. 5) and continue to increase after the mask has been removed and find a new steady state with higher concentration. For IL-25, there is a delayed response to mask application, whereby notable upregulation is observed ~50 min after the mask is removed. This switch-like behaviour is not fully captured by the model.

**Fig. 6.**
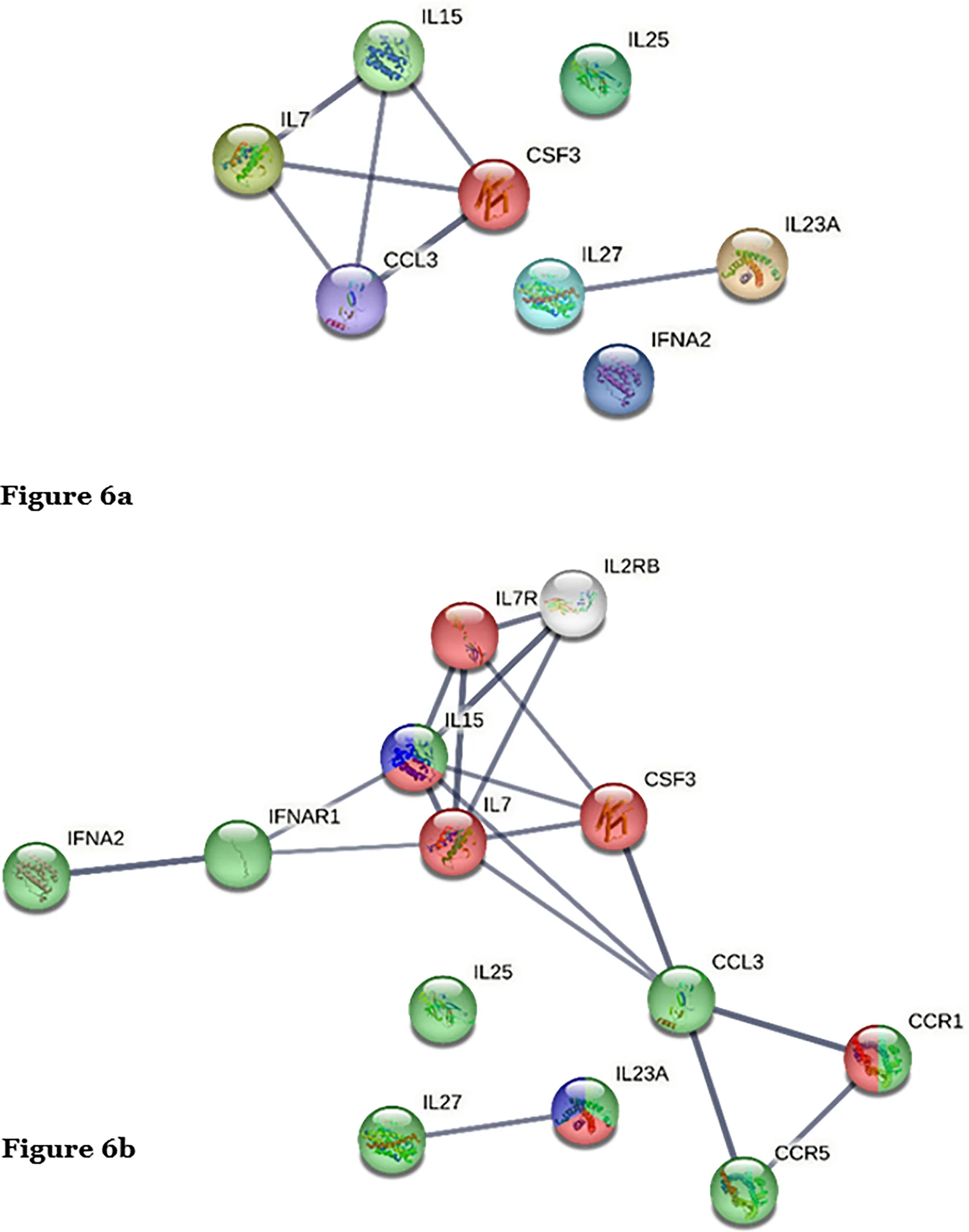
Protein network analysis of the 8 inflammatory proteins of most importance for the later timepoints. (a) The protein interaction network showing the later timepoints (40, 60, 120, 180 min) after loading in skin. The model has a high confidence interval (0.70), PPI enrichment value<0.0001. (b) The extended pathway analysis of the inflammatory response in later timepoints. Biological processes according to colours: Green: defence response (false discovery rate (FDR) *p-*value<0.0001), blue: positive regulation of haemopoiesis (FDR *p-*value<0.0001) and red: positive regulation of tissue remodelling (FDR *p*-value =0.0191). The model has a high confidence interval (0.70), PPI enrichment value<0.001.

The late responding proteins were further analysed to explore interactions and biological processes that they were involved in. The network interaction model showed a significant protein–protein interaction (PPI) enrichment value (<0.001) as illustrated in **Fig. 6**a. To explore larger connections also for the later time points, associated proteins were added into an extended network (Fig. 6b). Six proteins were added, namely, C-C chemokine receptor type 5 (CCR5), C-C chemokine receptor type 1 (CCR1), interleukin-7 receptor subunit alpha (IL7R), interleukin-2 receptor subunit beta (IL2RB), interferon alpha/beta receptor 1 (IFNAR1) and interferon alpha-2 (IFNA2). This extended pathway analysis, Fig. 6b, showed further interactions and biological processes, namely, defence response (FDR *p*-value <0.0001), positive regulation of haemopoiesis (FDR *p*-value <0.0001) and positive regulation of tissue remodelling (FDR *p*-value =0.0191).

Visible erythema at the nasal bridge directly after the NIV therapy were seen in 7 patients (64%), but only 1 patient had a persistent erythema up to 60 min. A supplementary OPLS-DA comparing erythema (*n*=7) and no erythema (*n*=4) at time-point 0 detected no differences in protein expression between the groups.

## DISCUSSION

This first-of-its-kind depth and temporal resolution uncover subtle immune dynamics in response to commonly used medical devices.

The distinct separation between protein expression in early timepoints (0 and 20 min) and the later timepoints (40, 60, 120 and 180 min) was validated by the mathematical models, which showed different cytokine dynamics between the 2 clusters. The cluster of early timepoints revealed that the inflammatory molecules increased during face mask application and decreased immediately after removal. The later responding cytokines increased slowly during mask application and continued to rise after removal, which may indicate ongoing tissue stress and subclinical inflammatory activity, even when no visible signs were present. This prolonged cytokine activity beyond mask removal highlights a window of ongoing tissue vulnerability that may precede visible skin damage.

Despite the relatively short duration of mechanical loading on the skin over the bridge of the nose (in mean 22 min), distinct inflammatory proteins and biological processes were associated with each group. Temporal cytokine profiling, combined with protein network analysis, revealed early engagement of angiogenic and remodelling pathways—despite no visible skin changes, suggesting that facemask use may initiate a silent wound-healing response.

The protein pathway analysis for the early group showed the biological process of *response to stress*, triggered by the mechanical load and shear on the skin caused by tight-fitting oronasal facemasks, potentially initiating inflammation to restore homeostasis disrupted by (often) external disturbances at the organismal or cellular level homeostasis (22). A prior study found that healthcare staff wearing protective respiratory facemasks had alterations in corneocytes over the nose bridge, similar to our findings, with increased immature cornified envelopes (CEs), indicating a stress response to preserve a compromised barrier (23).

Surprisingly, the protein network in the early group also showed biological processes of *response to wounding, angiogenesis involved in wound healing and cell migration involved in sprouting angiogenesis*, despite the relatively short duration of mechanical load. However, a majority of study patients (64%) had visible erythema directly after the removal of the facemask, indicating an inflammatory or vascular (post occlusive reactive hyperaemia) skin reaction. The regulation of angiogenesis and branching involved in blood vessel morphogenesis are late responders to skin damage and aims to restore oxygen and nutrition supply in tissue and to promote tissue repair (24). Furthermore, hypoxia induces the expression of VEGF-A (involved in the biological processes in the result) that promotes new blood vessels to form and thereby restore oxygen in the tissue, and are a crucial phase in wound healing (25). Involvement of these specific biological processes may imply that the short duration of the NIV mask therapy is still enough to cause microvascular damage and ischaemia. The nasal bridge, due to its bony prominence and limited soft tissue coverage, is susceptible to compression from mask loads, potentially exceeding capillary closing pressure and causing ischaemia and tissue damage.

In the group of later responders (40, 60, 120 and 180 min), the protein network analysis revealed the biological process of *defence response*, indicating a reaction to injury (22). The upregulated IL-25 plays an important role in the skin’s defence response to injury, by promoting keratinocyte proliferation and epidermal thickening (26). Corneocytes, the differentiated dead keratinocytes that constitute the stratum corneum, are defined by a cornified envelope and a keratin-filled interior, linked by corneodesmosomes. This structure is crucial for the stratum corneum’s mechanical strength and barrier function (27). In a prior study, the levels of corneodesmosomes were increased at the loaded skin site after respiratory masks vs control site, indicating that corneocytes might have an important role for skin integrity (23).

The protein network analysis also identified the biological process of *positive regulation of haemopoiesis*, that are activated by inflammation and aims to ensure the production of blood cells for efficient oxygen transport to tissues (28). Further, a *positive regulation of tissue remodelling* was identified which aims to restore tissue function and structure after damage (29). IL-15 and IL-23 (identified in our network analysis) play an important role as facilitators for skin tissue repair by enhancing natural killer cells, removing damaged cells (30), and stimulating proinflammatory cytokines (31). Our findings suggest that later responding inflammatory molecules continues to increase after removal of the NIV-mask, and that tissue remodelling and regulation of haemopoiesis occurs even in absence of visible erythema.

In a previous study by the authors investigating cytokine responses following combined chemical and mechanical loading of the skin (32, 33), substantial variability in biomarker concentrations was observed, particularly among low-abundance cytokines. Although total protein levels ranged from 23 to 130 μg/mL, normalization of inflammatory markers to total protein did not consistently reduce relative variability. For IL-1α, the coefficient of variation (CV) decreased slightly at one time point (68% vs 66%), but was similar or higher at others (82% vs 89%, 62 % vs 62%, and 60 % vs 76%) Fig. S3 and Table SII. These findings indicate no consistent improvement in variability following total protein normalization, see Appendix 1 for further details.

Accordingly, total protein was not analysed in the present study. Instead, the focus was placed on temporal changes in cytokine levels, which were considered more relevant than absolute secretion rates for capturing the dynamic inflammatory response. Moreover, similar total protein levels have been reported across different conditions, including stage 1 pressure ulcers (13) and controlled experimental skin loading (34). It should be noted, however, that the present study sampled proteins from the nasal bridge, whereas the referenced studies primarily assessed the sacral region, which may contribute to differences in local biomarker expression.

### Strengths and limitations

There are some study methodological limitations in this study. Firstly, the exploratory nature of the study and its small population size prevent generalization of the results. However, the multivariate analysis does not require large study populations to be robust, and this study contributes to the current knowledge gap by showing time-dependent changes in the early inflammatory response following mechanical loading to the skin. Secondly, although Sebutape® is a validated and safe method, it exclusively collects proteins from the skin’s surface, which may not accurately represent the physiology of deeper tissues.

Finally, the duration of mechanical loading in skin varied from 9 to 41 min (mean 22 min), which influences the inflammatory response. However, this observational study reflects real-life clinical application and the standard use of orofacial masks. Validation in larger cohorts will be essential to translate these insights into practical tools for early intervention and skin health monitoring.

### Conclusion

This explorative study presents the first comprehensive temporal profiling of local inflammatory responses in skin sebum following the standard use of oronasal facemask.

Importantly, our protein network analysis revealed activation of biological processes related to angiogenesis, defence response, regulation of cell death and tissue remodelling, despite the absence of visible skin changes. These findings suggest the presence of subclinical microvascular injury and early tissue stress, filling a critical gap in our understanding of how routine medical devices may silently impact skin integrity.

By uncovering the timing and complexity of inflammatory responses to facemask use, this study advances our understanding of pressure ulcer pathophysiology and opens new avenues for development of point-of-care biosensors for early detection and prevention of pressure ulcers.

## Data Availability

All relevant data are included in the manuscript, tables, and figures. The data that support the findings of this study are available on request from the corresponding author. The patient data is not publicly available due to privacy or ethical restrictions. Aggregated data (mean and standard error the mean), as well as all code and equations for the model simulations are provided in our GitHub repository https://github.com/willov/pressure-inflammation with a permanent copy available at Zenodo (DOI: 10.5281/zenodo.15187843).

## Supplementary material

Appendix 1.

Table SI.
