# Supplementary material for: Temporal Inflammatory Profile in Skin Surface after Oronasal Mask Application
Source: Acta Derm Venereol. 2026 Aug 31;106:0159. doi: 10.2340/actadv.v106.adv-2025-0159 (PMC13527610; doi:10.2340/actadv.v106.adv-2025-0159)
Supplement: Supplementary file 1 [file ActaDv-106-0159-s0001.pdf]

Supplementary description of total protein measurements from Sebutape samples on healthy volunteers during a lab-based repeated measures evaluation of simulated skin inflammation*

*Data from Abiakam NS, Jayabal H, Abbas S, Filingeri D, Bader DL, Worsley PR. The Effects of Incontinence Pad Application on Loaded Skin With Reference to Biophysical and Biochemical Parameters: An Exploratory Cohort Study Using a Repeated-Measures Design. J Wound Ostomy Continence Nurs. 2023 Nov-Dec 01;50(6):512-520. doi: 10.1097/WON.0000000000001032.

In a previous study (33), the temporal inflammatory response of the skin to synthetic urine (S-urine) exposure and mechanical loading was investigated in a cohort of healthy volunteers (n = 12). Three skin sites were identified in the sacral region of each participant: one exposed to a dry incontinence pad (site 1), one exposed to an S-urine–containing incontinence pad (site 2), and a third unexposed control site (site 3).

Baseline measurements were obtained prior to exposure. The pads were then applied, and the sites were left unloaded for 60 minutes. This was followed by 60 minutes of mechanical loading. Skin surface samples were collected using Sebutape at four predefined time points: (i) baseline (0 minutes), (ii) after 60 minutes of pad exposure (60), (iii) after an additional 60 minutes of loading (120), and (iiii) after a 30-minute recovery period (150 minutes from baseline). Substantial variability was observed in biomarker concentrations across the sampled skin sites, both within and between analytes. High-abundance cytokines, such as IL-1α and IL-1RA, showed wide concentration ranges (Figure S3), whereas several low-abundance markers (e.g., IL-6, IL-8, IFN-γ, and TNF-α) were often detected at levels close to the lower limit of the assay's calibration curve, indicating increased uncertainty in their quantification.

Total protein (TP) concentrations ranged between 23 and 130 μg/mL, reflecting considerable variation in the amount of material collected by Sebutape, illustrated in Figure S3. However, normalization of inflammatory biomarkers to TP did not improve the interpretation of the data or reduce variability across samples, table S2. This suggests that TP did not adequately account for differences in sampling efficiency or biological variability in this context.

Based on these findings, TP was not considered a meaningful normalization factor, and therefore, it was not included in the present study.
