# Supplementary material for: Temporal Inflammatory Profile in Skin Surface after Oronasal Mask Application
Source: Acta Derm Venereol. 2026 Aug 31;106:0159. doi: 10.2340/actadv.v106.adv-2025-0159 (PMC13527610; doi:10.2340/actadv.v106.adv-2025-0159)
Supplement: Supplementary file 2 [file ActaDv-106-0159-s0002.pdf]

SUPPLEMENTARY TABLES

Table SI. The 17 inflammatory proteins of most importance for separation between time intervals. Data are expressed as absolute number (pg/ml) or median and min-max. VIP > 1.0 and p(corr) > 0.4 were considered as significant. Mann-Whitney U test was conducted to test significance level of comparison between group 1 (0-20 min) and group 2 (40-180 min). A p-value < 0.05 was considered as significant.

**Abbreviations**	**Protein name**	**VIPpred**	**p(corr)**	**Group 1, pg/ml median (min-max)**	**Group 2, pg/ml median (min-max)**	**p-value**
**IFN-α2**	Interferon alpha-2	2.22	-0.72	0.27, 0.003- 0.486	0.41, 0.16- 0.73	<0.0001*
**IL-1α**	Interleukin-1 alpha	1.78	0.58	393.6, 63.33-1592	188.9, 37.75-590.8	0.0033*
**IL-1β**	Interleukin-1 beta	1.62	0.53	10.04, 2.03- 47.90	4.84, 1.14- 29.34	0.0023*
**IL-25**	Interleukin-25	1.55	-0.51	0.17, .00- 1.01	1.046, .00- 3.01	0.0007*
**CSF3**	Granulocyte colony-stimulating factor	1.55	-0.50	0.11, .00-0.93	0.41, .00- 108.8	0.0043*
**CHI3L1**	Chitinase-3-like protein 1	1.54	0.50	4472, 1145-13861	3988, 940-12507	0.0515
**CSF1**	Macrophage colony-stimulating factor 1	1.53	0.50	0.35, 0.04-1.21	0.18, 0,02-7.54	0.0012*
**VEGFA**	Vascular endothelial growth factor A	1.50	0.49	16.20, 3.18- 54.01	9.20, 2.57- 667.1	0.0022*
**CX3CL1**	Fractalkine	1.49	0.49	70.36, 32.37-160.3	50.68, 4.69-3330	0.0453*
**IL-7**	Interleukin-7	1.47	-0.48	0.25, .00- 0.60	0.41, 0.04- 23.15	0.0094*
**IL1RN**	Interleukin-1 receptor antagonist protein	1.46	0.47	4715, 3560-5076	4269, 1971-4766	<0.0001*
**IL-2RA**	Interleukin-2 receptor subunit alpha	1.43	0.46	6.02, .00- 26.40	.00, .00- 24.56	0.0003*
**IL-27**	Interleukin-27	1.41	-0.46	11.46, 2.97-23.64	14.73, 8.60-32.25	0.0211*
**IL-23**	Interleukin-23	1.38	-0.45	0.36, .00- 1.29	0.74, .00-4.88	0.0367*
**IL-9**	Interleukin-9	1.37	0.44	0.10, .00-0.44	.00, .00-0.39	0.0015*
**CCL3**	C-C motif chemokine 3	1.35	-0.44	0.27, .00- 6.18	3.45, .00-9.74	0.0086*
**IL-15**	Interleukin-15	1.26	-0.41	0.24, .00-0.79	0.37, 0,04-5.65	0.0023*

Table SII. Coefficient of variation (CV) of inflammatory markers with and without total protein (TP) normalization. CV (%) was calculated as the standard deviation divided by the mean × 100 for Interleukin-1α at each sampling time point.

TIMEPOINT	IL-1α CV (%)	IL-1α/TP CV (%)
**0 MIN**	81.73	88.86
**60 MIN**	68.54	66.61
**120 MIN**	62.32	61.89
**150 MIN**	59.54	76.35
