# Supplementary material for: Temporal Inflammatory Profile in Skin Surface after Oronasal Mask Application
Source: Acta Derm Venereol. 2026 Aug 31;106:0159. doi: 10.2340/actadv.v106.adv-2025-0159 (PMC13527610; doi:10.2340/actadv.v106.adv-2025-0159)
Supplement: Supplementary file 3 [file ActaDv-106-0159-s0003.pdf]

SUPPLEMENTARY FIGURES

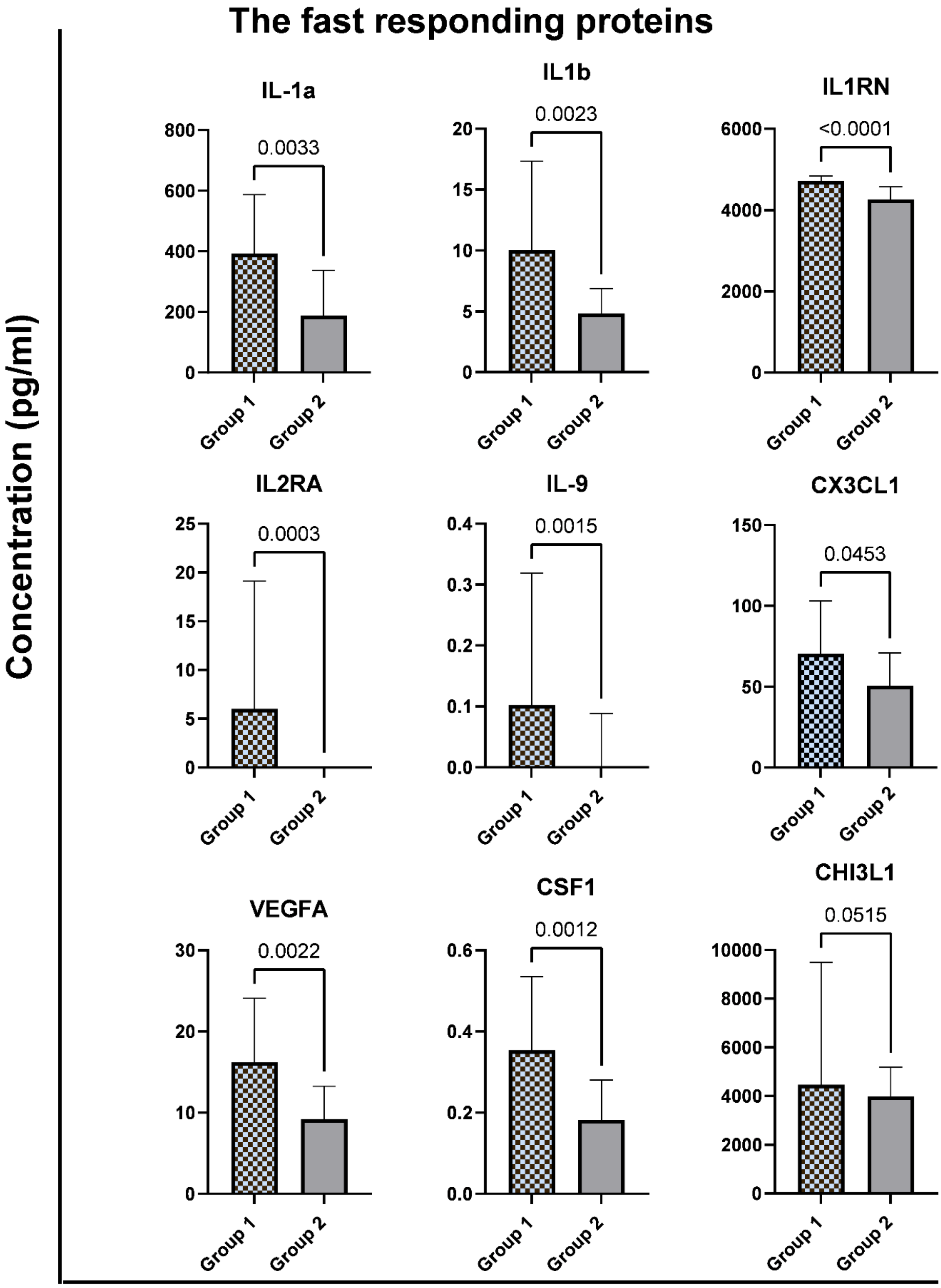

Fig. S1. Barplots of the fast-responding proteins. Mann-Whitney U test for comparison of median value of protein expression between the timepoints. Group 1=0+20 minutes, group 2= 40-180 minutes. P-value <0.05 was considered as significant.

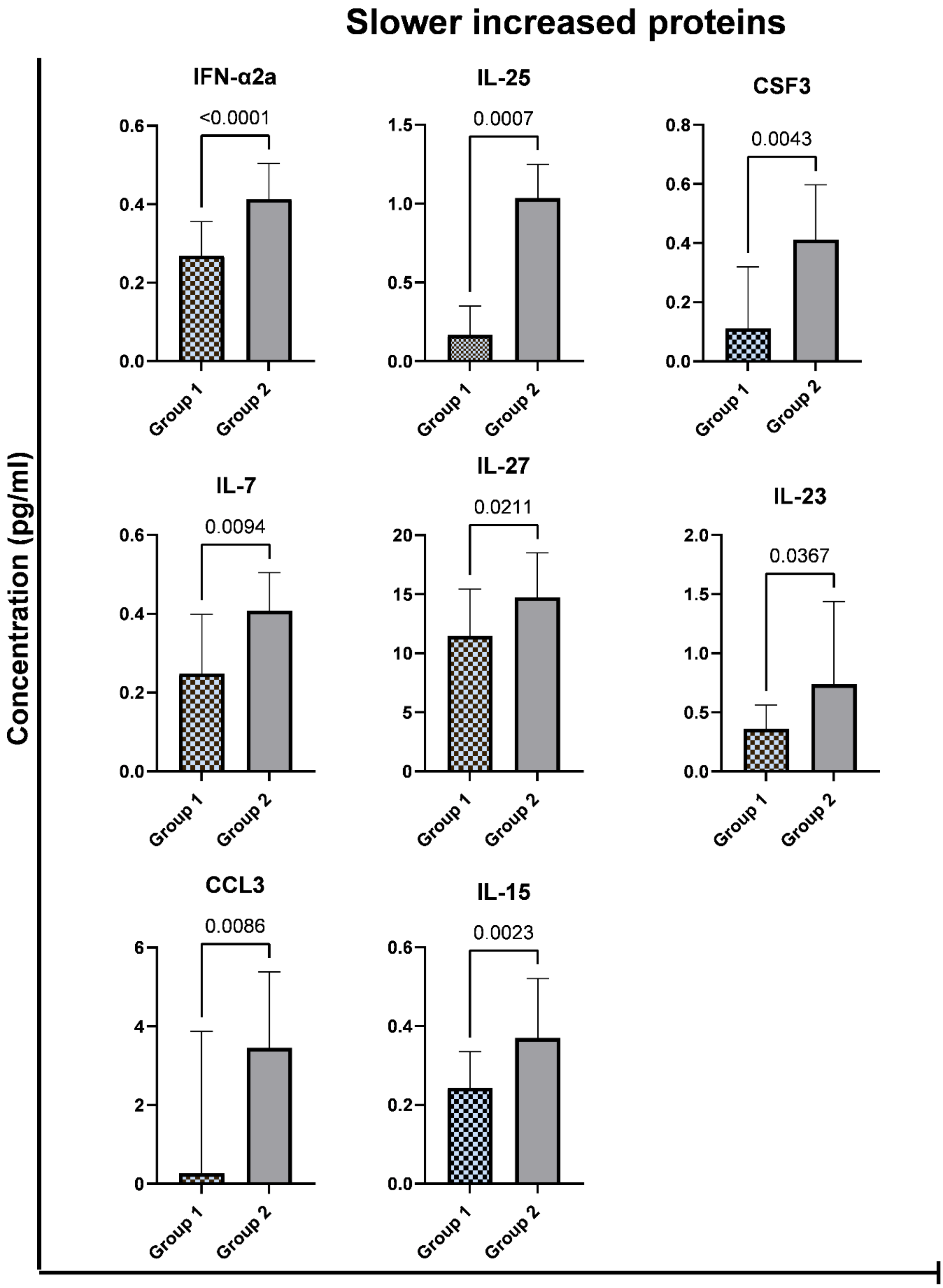

Fig. S2. Barplots of the slower increased proteins. Mann-Whitney U test for comparison of median value of protein expression between the timepoints. Group 1=0+20 minutes, group 2= 40-180 minutes. P-value <0.05 was considered as significant.

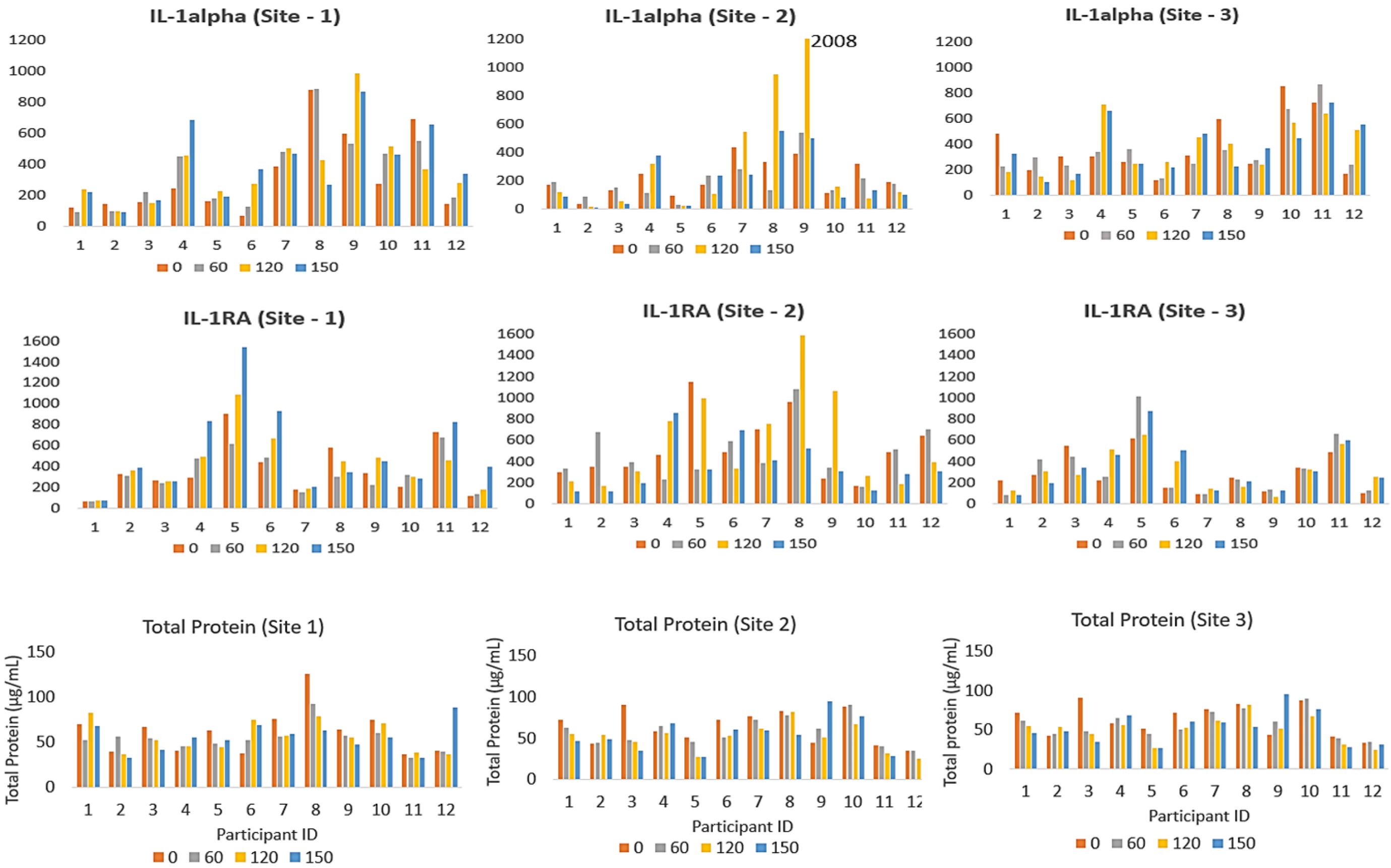

Figure S3. Absolute concentrations of IL-1α, IL-1RA and total protein at the three sacral sites and the temporal changes. Figure replicated with permission from the authors (32). Site 1= Dry pad, Site 2= Pad with S-Urine, Site 3= Control area. Mechanical loading applied between timepoint 60-120.
